# Shape Memory Alloys in Textile Platform: Smart Textile-Composite Actuator and Its Application to Soft Grippers

**DOI:** 10.3390/s23031518

**Published:** 2023-01-30

**Authors:** Jin Shin, Ye-Ji Han, Ju-Hee Lee, Min-Woo Han

**Affiliations:** Department of Mechanical, Robotics and Energy Engineering, Dongguk University, 30 Pildong-ro 1 gil, Jung-gu, Seoul 04620, Republic of Korea

**Keywords:** smart materials, shape memory alloys (SMAs), textile actuators, soft grippers, soft robotics

## Abstract

In recent years, many researchers have aimed to construct robotic soft grippers that can handle fragile or unusually shaped objects without causing damage. This study proposes a smart textile-composite actuator and its application to a soft robotic gripper. An active fiber and an inactive fiber are combined together using knitting techniques to manufacture a textile actuator. The active fiber is a shape memory alloy (SMA) that is wire-wrapped with conventional fibers, and the inactive fiber is a knitting yarn. A knitted textile structure is flexible, with an excellent structure retention ability and high compliance, which is suitable for developing soft grippers. A driving source of the actuator is the SMA wire, which deforms under heating due to the shape memory effect. Through experiments, the course-to-wale ratio, the number of bundling SMA wires, and the driving current value needed to achieve the maximum deformation of the actuator were investigated. Three actuators were stitched together to make up each finger of the gripper, and layer placement research was completed to find the fingers’ suitable bending angle for object grasping. Finally, the gripping performance was evaluated through a test of grasping various object shapes, which demonstrated that the gripper could successfully lift flat/spherical/uniquely shaped objects.

## 1. Introduction

Soft actuators open up new possibilities for robotics by solving challenges with a novel approach to mechanical motions that are difficult to accomplish with conventional actuators [1]. Since soft actuators are comprised of soft and/or extensible materials (e.g., silicone rubber and textiles) with a high energy-absorbing capability, these actuators are safer to operate than rigid actuators and are suitable for application in wearables and medical devices [2,3]. In addition, due to their simple structure, soft actuators have distinct advantages for manufacturing, actuation, and repair [4].

Soft actuators can be composed of intelligent materials that respond to external environmental changes, such as electricity, light, pH, and temperature, which include reactive gels/hydrogels, ionic polymer metal composites, conductive polymers, carbon nanotubes/graphene, and dielectric elastomers [5,6,7,8]. Araromi et al. utilized pre-stretched dielectric elastomer actuators to create a rollable actuator [9]. Bhattacharya et al. developed a compliant two-jaw gripper using an ion-exchange polymer–metal composite (IPMC) [10]. Fan et al. introduced flexible double-layer electrothermal actuators made from polyimide (PI) film bonded to graphite paper [11]. Yang et al. developed a light-driven autonomous carbon-nanotube-based bimorph actuator, which performs a continuously self-oscillating motion in a wavelike fashion, mimicking the human sit-up motion [12]. Cheng et al. reported intelligent bilayer-type fluorescence hydrogels, which generate a bending motion in response to temperature and pH [13].

Shape memory alloy (SMA), an intellectual material, is a distinctive shape-memorizing material that remembers its trained form when exposed to heat, which has become a major interest in producing soft actuators due to its unique characteristics and potential for engineering applications [14]. SMA has excellent mechanical properties such as a high corrosion and abrasion resistance, a long fatigue life, a great biocompatibility, pseudo-elasticity, a high actuation-energy density, and a light weight [15,16]. Furthermore, SMA has a relatively short activation time and high actuation-generated stress compared to other materials, making it suitable for developing actuators [17]. Koh et al. fully utilized these advantages to produce a 68-milligram at-scale robotic insect that jumps on the surface of water using SMA [18]. New types of SMA actuators can also be created in combination with other materials, such as polymer actuators based on SMA tendon wires that are capable of generating bending motions to grasp a variety of materials and shapes [19,20,21].

A textile structure is flexible in all dimensions, enabling an extensive range of movement in a limited space [22]. A textile structure conforms naturally to the contours and bends of an object, maintains its deformed shape, and easily returns to its original configuration by external force [23]. Moreover, a textile structure has a uniform and smooth surface and is capable of combining multiple materials in the form of thread, which is useful for wearable applications [24]. Taking advantage of these unique and advantageous characteristics, many researchers have been studying soft actuators and sensors based on a textile structure [25]. Schaffner et al. developed robotic soft actuators with programmable bioinspired architectures, using a 3D-printing technique to produce a textile structure [26]. Phan et al. introduced fluid-driven smart textiles formed from soft artificial muscle fibers produced by a knitting and weaving technique, which is capable of creating a four-legged structure, a fabric butterfly, and a flower [27]. Proesmans et al. proposed a fully wireless, highly flexible, light, and modular version of a piezoresistive smart textile sensor, sufficient for classification tasks [28]. Yu et al. developed a soft capacitive microfiber sensor that can be woven seamlessly into textiles for strain measurement [29].

There have been a number of studies to develop textile actuators using SMAs, generating a significant synergy effect between a textile structure and an SMA material. Textile actuators using SMAs feature a light weight, silent operation, a simple production process, and low-cost manufacturing [30]. An intelligent SMA wire has 446 J/kg specific strength when stretched, and, when it is knitted to form a textile structure, it can create an autonomous soft actuator [31]. Han et al. developed an SMA-based textile morphing flower that blooms under an electric current and confirmed its various modes of deformation [32]. Mersch et al. presented an SMA-based integrated soft sensor–actuator system with a bending angle of 270° [33].

**Figure 1:**
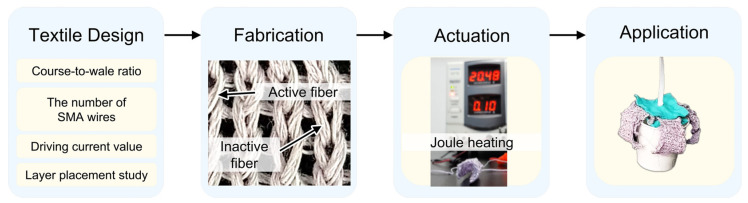
Methodology flow chart illustrating the major milestones of this work.

In this study, we introduce a novel method for soft morphing structures based on loop-linked structures with functional fibers, which allows for the creation of three-dimensional morphologies. A morphing structure was fabricated based on the design process of loop patterning, followed by an analysis of the current-induced three-dimensional volumetric transformation. The soft morphing structure was constructed by knitting SMA-based functional fibers and conventional yarn together. As there has been no study focused comprehensively on the design and operating configuration of SMA textile actuators, we conducted an investigation into the course-to-wale ratio of the knitting structure, the number of bundling SMA wires, and the current value for actuating. Moreover, by not only utilizing a single-layered SMA knitted textile, the performance of single-/double-/triple-layered structures was investigated and compared to each other to generate a suitable bending angle. A soft morphing textile gripper was introduced as an application of the proposed soft morphing structure, and its performance was evaluated through a grasping test. The gripper successfully lifted flat/spherical/uniquely shaped objects, confirming that the aforementioned configuration study and layer placement study have potential for practical use. Figure 1 briefly depicts the milestones of this work, from the textile design to the application as a soft robotic gripper.

## 2. Materials and Methods

### 2.1. Design and Fabrication of the Soft Morphing Textile Actuator

The soft morphing textile actuator is made by combining active fiber and inactive fiber with knitting technique, which features as soft and compliant a structure as knitted clothing does.

**Figure 2:**
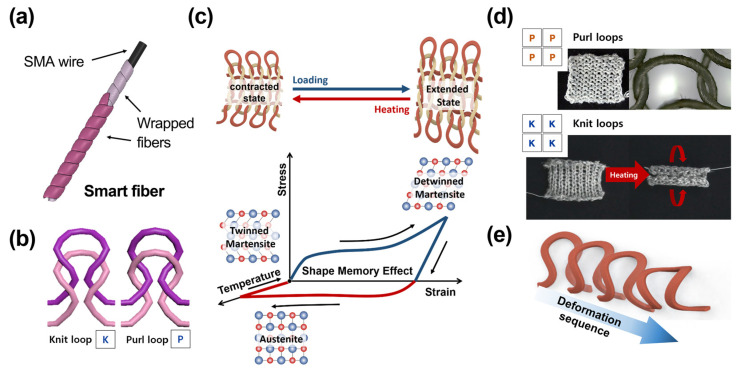
(**a**) The active fiber consists of the SMA wire wrapped by polyester fiber. (**b**) The loops are divided into knit (K) and purl (P) loops. If the top half of a loop is behind the previous loop, it is a K; if it is in front, it is a P. (**c**) Phase transformation of SMA wires in knitting platform. (**d**) The stockinette stitch of the textile actuator and its actuation under heating. (**e**) The driving mechanism of a knitted SMA wire loop with increasing temperature during the phase transformation.

An SMA wire was used as the driving source. The SMA, one of the most commonly used thermally responsive materials, undergoes its phase transition from martensite phase to austenite phase with the application of heat. This phenomenon is known as shape memory effect (SME) [30]. There are two major phases in the SMA: the martensite phase from martensite start temperature ($M_{S}$) to martensite final temperature ($M_{f}$) and the austenite phase from austenite start temperature ($A_{S}$) to austenite final temperature ($A_{f}$). In the austenite phase, the SME is activated, and the SMA returns to its trained shape. When it returns to a low temperature (e.g., room temperature), the SMA wire returns to the martensite phase, where it can move freely out of its trained form. The active fiber changes its length under certain amount of electric current. As the active fiber, Ni-Ti SMA wire (55 wt% Ni and 45 wt% Ti) wrapped with polyester fiber is adopted (Figure 2a). This wrapping process prevents short-circuiting between SMA knitted fibers and increases friction coefficient, which improves actuating performance by avoiding fiber slip [32]. By strengthening frictional force formation even more, the actuator’s capacity for matrix retention is improved. The SMA wire core has diameter of 200 μm, and it shortens with shrinkage rate of 6–8% above the phase transition temperature (68 °C) [34].

On the other hand, the inactive fiber does not respond to any thermal or electrical stimulus; as it is solely capable of passive-way transformation. Conventional knitting yarn was used as the inactive fiber. The diameter of knitting yarn is 1.5 mm, and its composition is 85/15 cotton/polyester.

As both active and inactive fibers are thin threads, they can be made into the form of fabric by textile manufacturing process. Knitting, which uses one or more yarns to create a continuous loop, is one of the most-used textile production techniques. Based on a loop head of two pairs of loops arranged above and below, it is possible to create two types of basic loops: a knit loop and a purl loop. For knit loops, the lower loop’s head is positioned behind the upper loop, but, for purl loops, the lower loop’s head is placed in front of the upper loop (Figure 2b) [35]. The looping architecture enhances elasticity of the textile. When holes are formed by looping, they can be widened or narrowed, while maintaining the whole knitting structure. Therefore, the actuator has an excellent structure-retention ability. Moreover, an SMA actuator produced by the knitting method gives a more significant potential for diversifying design than a straight-lined SMA wire actuator [32].

The actuator can be activated by raising the temperature, which can be accomplished with a heating gun or Joule heating with electrical current. Figure 2c shows the phase transformation of the SMA in the knitting platform. Two parallel edges bend toward the center in response to external heat because the actuator has the stockinette stitch pattern, which has an asymmetric geometry causing curls in one direction (Figure 2d) [32]. Figure 2e shows the driving mechanism with increasing temperature during the phase transformation of the knitted SMA wire, which generates a curling motion.

### 2.2. Theoretical Analysis

There is a three-dimensional and phenomenological model for the constitutive SMA model [36,37,38,39,40]. The shape memory effect of the SMA, which occurs under temperature change between the martensite and austenite phase, exhibits a large residual strain. From Liang’s constitutive model for the SMA behaviors, the constitutive relation is given, under the assumption that all material functions are constants:

(1) $$\sigma_{SMA}-\sigma_{0,SMA}=\beta \left(\varepsilon_{SMA}-\varepsilon_{0,SMA}\right)+\Omega \left(\xi -\xi_{0}\right)+\varphi \left(T-T_{0}\right)$$

where β is the modulus function of the SMA material, Ω is the transformation tensor, φ is the thermal coefficient related with the SMA expansion, and ξ is the martensite fraction factor. Assuming the phase transformation from the initial conditions of $\left(\sigma_{0,SMA}=\varepsilon_{0,SMA}=\xi_{0}=0\right)$ to the final conditions of ($\sigma_{SMA}=0,\;\varepsilon_{SMA}=\varepsilon_{L,SMA},\;\xi =1$) occurs by perfectly transforming from austenite to detwinned martensite (ξ=0 to ξ=1), the transformation tensor relationship is derived as

(2) $$\Omega =-\varepsilon_{L,SMA}\beta$$

The empirical model for the transformation from martensite to austenite can be described by

(3) $$\xi =\frac{\xi_{0}}{2}\left\{\cos\left[\alpha_{A}\left(T-A_{S}-\frac{\sigma_{SMA}}{C_{A}}\right)\right]+1\right\}\;\;\mathrm{for}\;C_{A}\left(T-A_{f}\right)<\sigma_{SMA}<C_{A}\left(T-A_{S}\right)$$

The material constants $C_{A}$ describes the relationship between temperature and the critical stress, which is assumed to be a continuously constant value over all ranges of temperature, and $\alpha_{A}$ is given as

(4) $$\alpha_{A}=\pi /\left(A_{f}-A_{S}\right)$$

**Figure 3:**
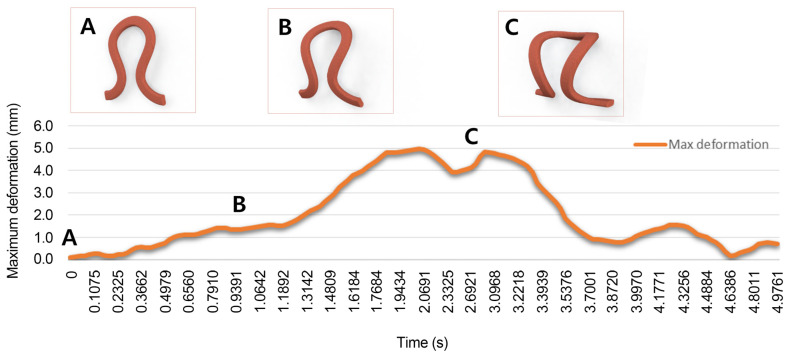
A theoretical modeling with simulation results of a unit loop of SMA wire; generation of a curling motion by increasing the temperature of the knitted wire. A is the original shape of the loop, B is the loop under deformation, and C is the loop at its maximum deformation.

During actuation, the actuator is assumed to present a circular geometry by holding the SMA wire at an original position, with the force concentration necessary for bending deformation regardless of the shear, torsional, and axial deformation. Figure 3 shows theoretical modeling with simulation results of a unit loop made of SMA materials. As a result, the knitted SMA wire generates a curling motion by increasing the temperature. Moreover, the material properties of SMA are presented in Table 1 [34].

## 3. Investigation into Design and Actuating Conditions of Textile Actuator

Prior to fabricating a textile gripper, the structural characteristics and operating conditions of the actuators should be investigated. In this section, we use an experimental approach to investigate the actuation characteristics of the textile actuator, which are dependent on the configuration of the textile and the current application.

### 3.1. Course and Wale Ratio

**Figure 4:**
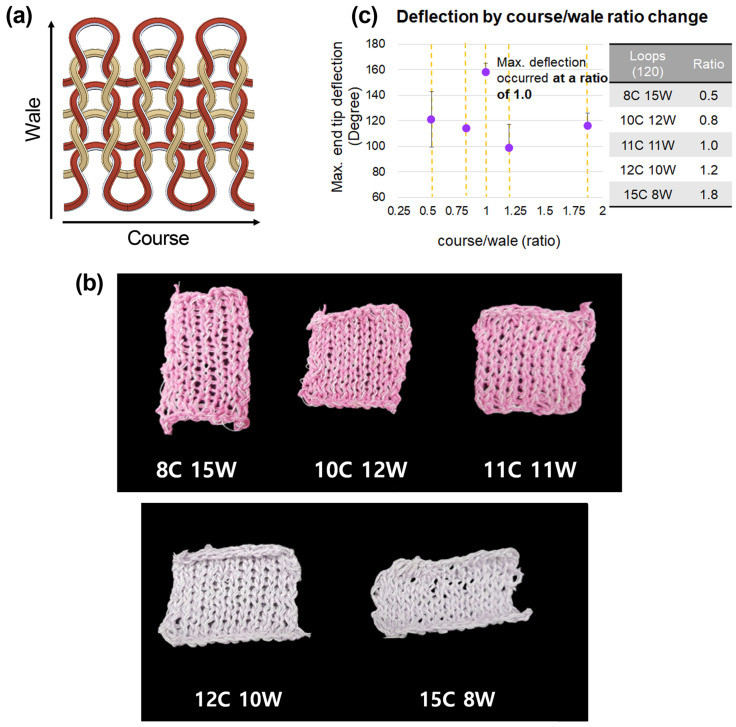
(**a**) Schematic diagram of knitted fabric. (**b**) The appearance of the actuator by course-to-wale ratio. (**c**) The deformations of actuators with different composition ratios of courses and wales.

In knitted textiles, courses refer to horizontally arranged loops, whereas wales refer to vertically arranged loops, as shown in Figure 4a [35]. Since the deformation in the wale direction is greater than that in the course direction due to the characteristics of the knitting structure, the actuating angle is expected to vary according to the course–wale ratio [32]. Therefore, we conducted an experiment to observe the deformation that is dependent on a different course-to-wale ratio. The current was increased stepwise from 0 to 0.16A. The number of courses and wales was adjusted to create about 120 loops, resulting in an SMA wire length of 175 cm. Figure 4b shows the appearance of the actuator by the course-to-wale ratio. The maximum end-tip deflection was obtained by measuring the angle between the end of the actuator that is affixed to the bottom surface and the other end tip of the actuator. As a result of the experiment, the actuator with a course-to-wale ratio of 1:1 showed maximum bending of 157 degrees on average (Figure 4c). Therefore, when the same current is applied, an actuator with a 1:1 course-to-wale ratio yields the highest deformation efficiency.

### 3.2. Number of Bundled SMA Wires

**Figure 5:**
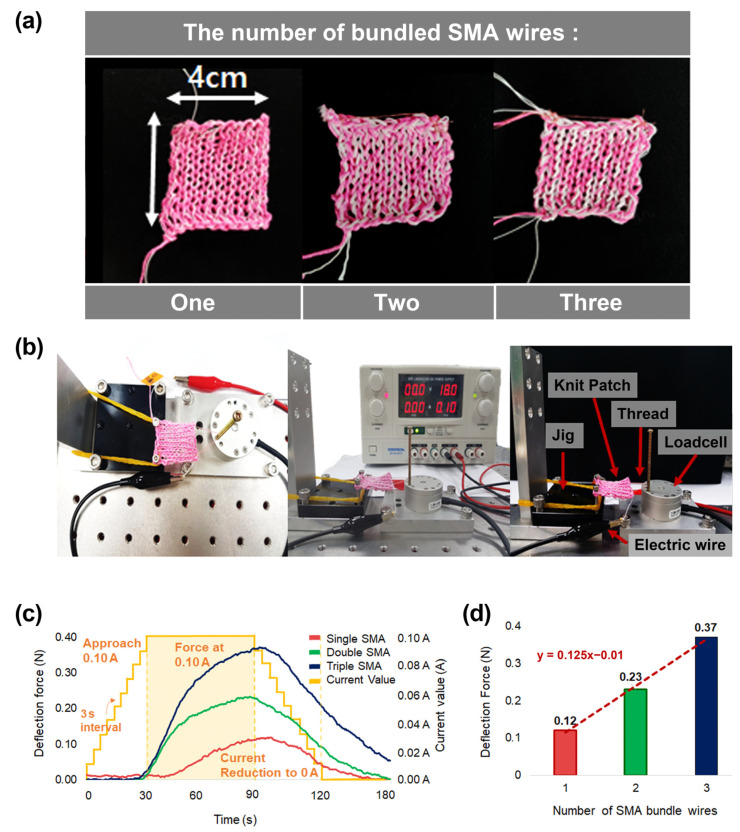
(**a**) Ten-course and ten-wale actuators with different numbers of SMA wires to create textile. (**b**) Test environment of force measurement. (**c**) Deformation force of textile actuator according to the number of SMA wires. (**d**) The rate of increase in force by increasing the number of SMA wires.

An additional SMA wire can increase actuation force; it is also possible to knit several SMA wires together during actuator production to meet specific requirements. We manufactured actuators with different numbers of bundled wires (1–3) and monitored the bending force of each actuator. All the actuators were 30 × 30 ${\mathrm{mm}}^{2}$ and had a course-to-wale ratio of 1:1 (Figure 5a). To measure the actuator’s exerting force, an edge of the actuator was fixed to the jig with bolts, and the opposite edge was connected to a thread. The thread was then hung on a nail, which was fixed to the load cell (Figure 5b). The current was increased from 0 to 0.10 A over 30 s, at 3 s intervals, maintained at 0.1 A for 60 s, reduced to 0 in intervals of 3 s, and then cooled for 30 s. Figure 5c shows the effects of the number of SMA wires on the force. The actuator with one SMA wire exerted an average force about 0.12 N in the X-axis direction, which was parallel to the loop of the actuator’s head-to-tail direction. Using two SMA wires, the average force increased by 1.92 times, to 0.23 N. The average force rose to 0.37 N when three SMA wires were used, an increase of an additional 1.6 times. Figure 5d shows that the maximum force in the X-axis direction of the actuator varies as the number of SMAs changes. This demonstrates that there is a linearly proportional relationship between the number of SMA wires and the magnitude of the force generated by the actuator.

### 3.3. Electric Power Input

**Figure 6:**
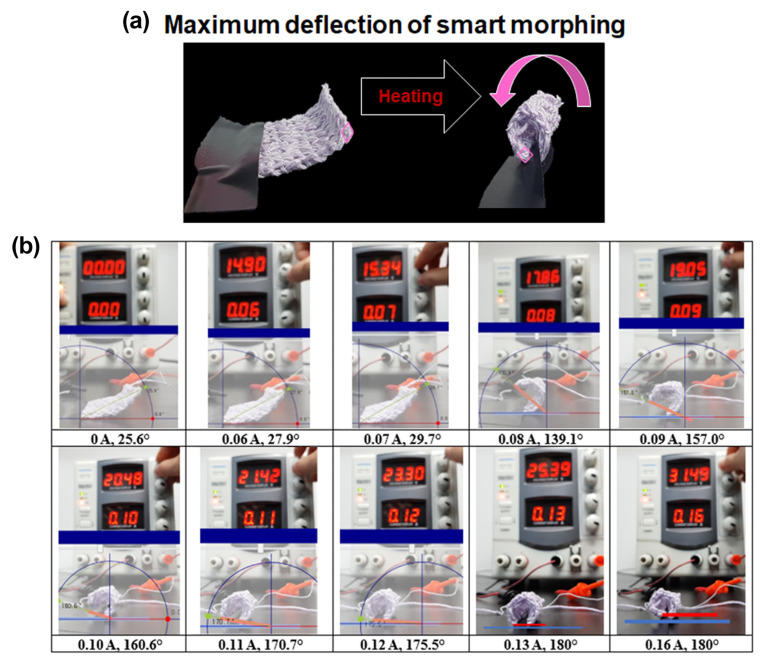
(**a**) The images of the actuator before and after actuation. (**b**) Experimental setup; measurement of the actuator’s deformation angle.

**Figure 7:**
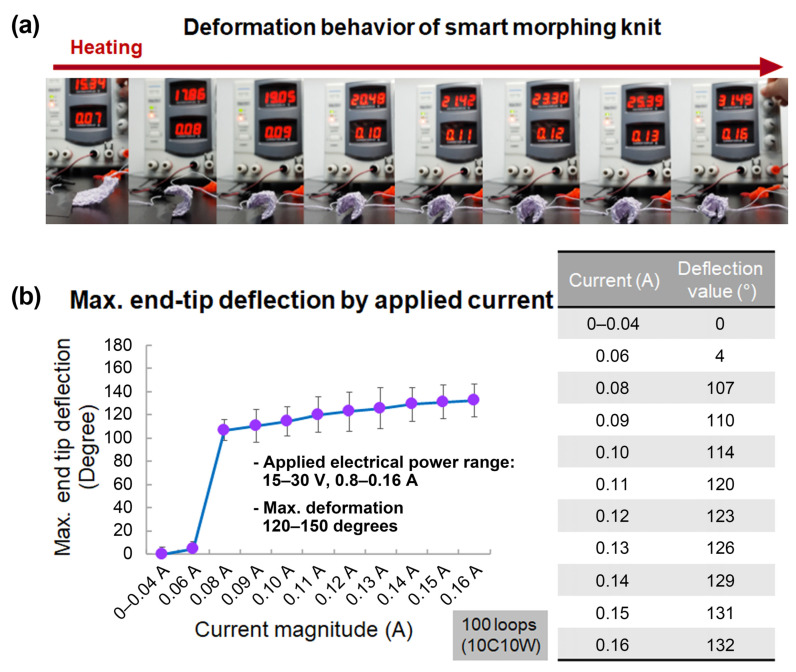
(**a**) The maximum deformation of a textile actuator according to the extent of current flow. (**b**) The maximum bending deformation angle of a 10-course and 10-wale actuator.

The SMA in the shape of a continuous loop undergoes a phase change, from martensite to austenite, under the influence of heating by an electric power input. Figure 6a shows the images of the actuator before and after actuation. If the magnitude of the current is too small, the actuator may not produce the desired deformation or may even fail to move at all. Conversely, an excessive current flow to the SMA wire can cause the SMA wire to overheat and lose its trained shape [41]. The actuator has a convergence value on deformation, so the displacement value does not grow continuously when the applied power is increased. One corner of the actuator fixed to the ground, the maximum bending angle of the opposite corner at a given current was measured (Figure 6b). Then, the end-tip deflection was recorded by subtracting the initial angle from the last angle. The actuator’s course-to-wale ratio was 1:1, and three bundles of SMA wires were used. We observed the maximum bending angle while increasing the current by 0.01 A in a stepwise manner, from 0 A to 0.16 A (Figure 7a). Figure 7b shows the results of the bending deformation of the actuator. At less than 0.08A, deformation hardly occurred; at 0.06 A, only 4 degrees were deformed on average. However, at 0.08 A or more, the actuator curled rapidly. Additionally, there was no discernible difference in the deformation angle, within 1.5%, for a current increase of 0.14 A or more.

## 4. Textile Gripper

### 4.1. Multi-Layered Structure

**Figure 8:**
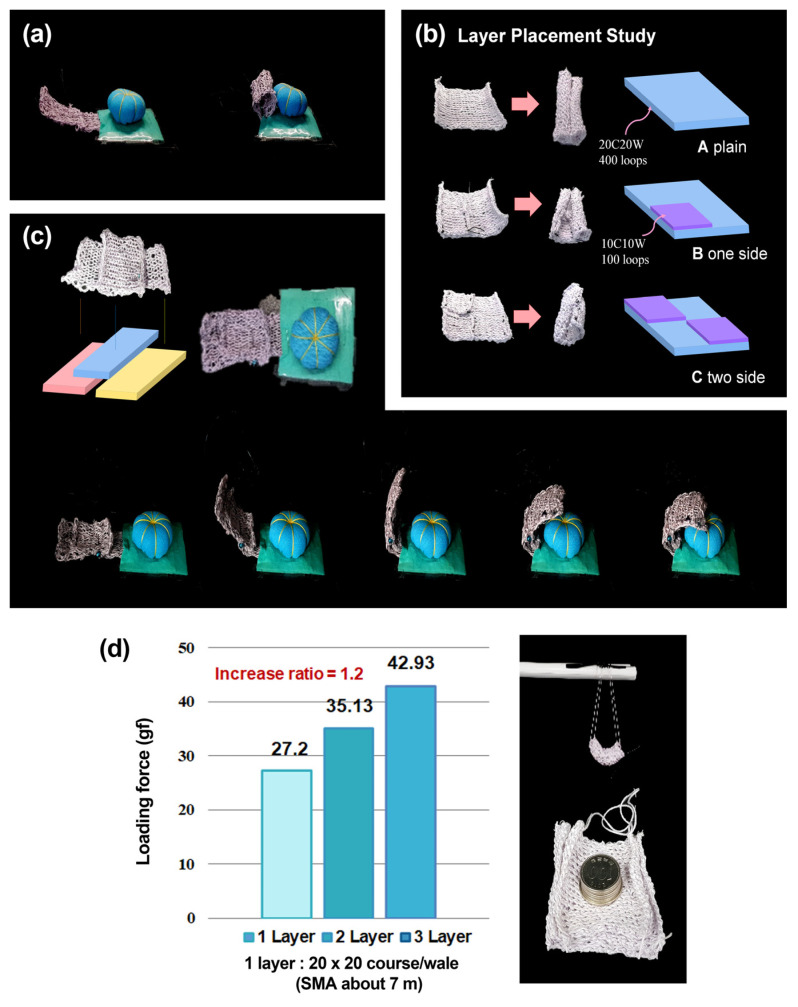
(**a**) The deflection of a single-layered finger; fully curled in. (**b**) The bending behavior when a 10-course and 10-wale actuator was placed on a 20-course and 20-wale actuator. (**c**) A gripper finger with double-layered structure and its verification of the functionality. (**d**) Coin-lifting experiment using several stacked 20-course and 20-wale actuators.

Knitted morphing textile actuators can be stitched longitudinally to form fingers, and each finger can be stitched to the corner of a square of fabric to form a textile gripper. The gripper can be driven by flowing current through each finger. Figure 8a shows the deflection when a finger is actuated. The finger is fully curled in, which is unsuitable for gripping objects. Therefore, it is necessary to improve the gripping performance through curvature control.

We solved the problem with layer placement. It was anticipated that, by stacking multiple layers, it would be possible to prevent the actuator from deforming abruptly and to produce a gentle curve. We conducted a layer placement study to demonstrate whether this is a feasible solution. A small actuator was placed on top of a large one, and deformation under heating was observed. In Figure 8b, ‘A’ has a single layer, which shows the original deformation with both corners fully curled in. ‘B’ has a small top actuator sewn to a part of bottom actuator. At the double-layered area, the extent of deformation is reduced, while the single-layered area still shows the fully curled in deformation. ‘C’ has two small actuators arranged diagonally; the bending angle over the entire actuator is reduced. As shown in Figure 8c, we applied the double-layered structure to a gripper finger and verified the functionality of the structure by checking the angle of the finger while grasping an object. At the double-layered region, there was a noticeable decrease in the curvature, so the end tip of the finger could reach the object, which showed an enhanced gripping performance.

Furthermore, a multi-layered structure increases the actuating force. We measured the weight that the actuator could withstand when several same-sized actuators were stacked (Figure 8d). When the actuator was in action, it curled inward, and the object could be fixed. In this weight-withstanding experiment, coins were stacked onto the actuator, and the heaviest weight that the actuator could support was recorded. As a result, the actuating force was increased in proportion to the number of layers. The increase ratio was 1.2. Moreover, even without current being supplied, the coins were held by the actuator, which suggests that an energy-saving actuating method could be implemented: once the actuator reaches its ideal deformation, the power supply could be cut while the actuator retains its deformed shape. Therefore, a multi-layer structure can adjust the curvature of the actuator and increase the actuating force.

### 4.2. Soft morphing Textile Gripper

**Figure 9:**
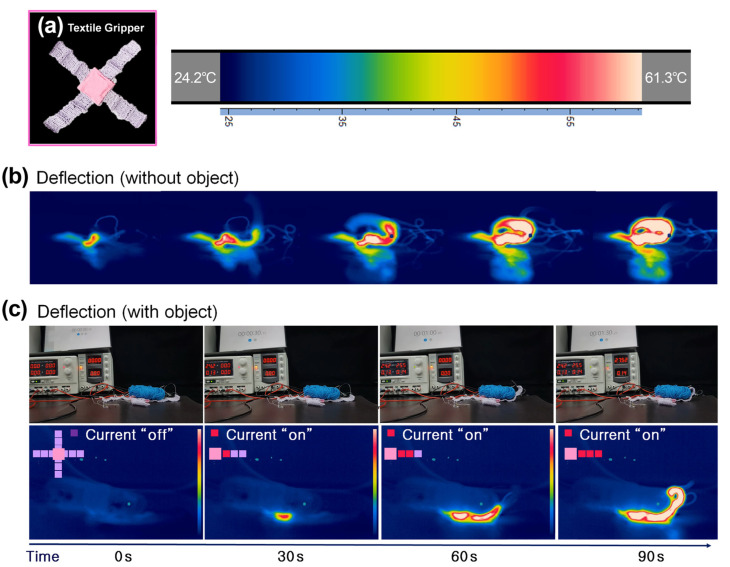
(**a**) Textile gripper consisting of textile actuators. (**b**) The stepwise deformation of the textile gripper without an object. (**c**) The stepwise deformation of the textile gripper with an object.

As shown in Figure 9a, we made a soft gripper with four fingers, with each finger consisting of three connected actuators. The individual actuators were insulated from each other, which enabled the stepwise actuation of the finger. The actuators’ course-to-wale ratio was 1:1, and the number of SMA bundle wires was three, in keeping with the design constraint study presented in Section 3. Figure 9b and c show the finger’s deformation under a current supply of 0.14 A to each actuator at intervals of 30 s. The entire deformation was recorded by a thermal camera (Video S1). In the absence of an object (Figure 9b), the finger was bent entirely in; when an object was present, the finger gently wrapped the object, and the end was bent like a claw to hold it (Figure 9c) [42].

**Figure 10:**
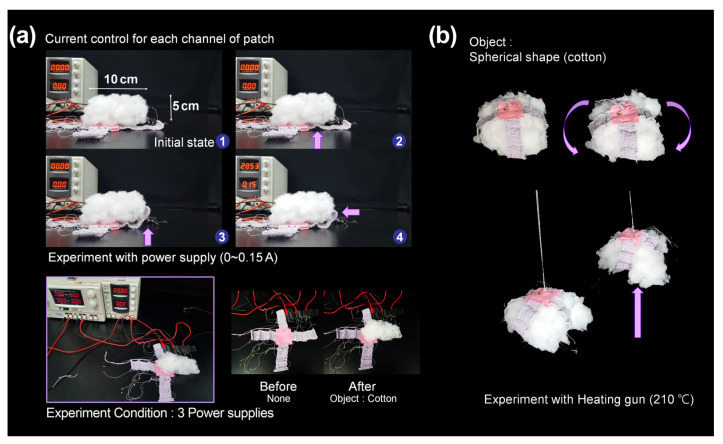
(**a**) Current control for each channel of textile actuators. (**b**) A bulky-shaped piece of cotton is gripped and lifted by the textile gripper.

**Figure 11:**
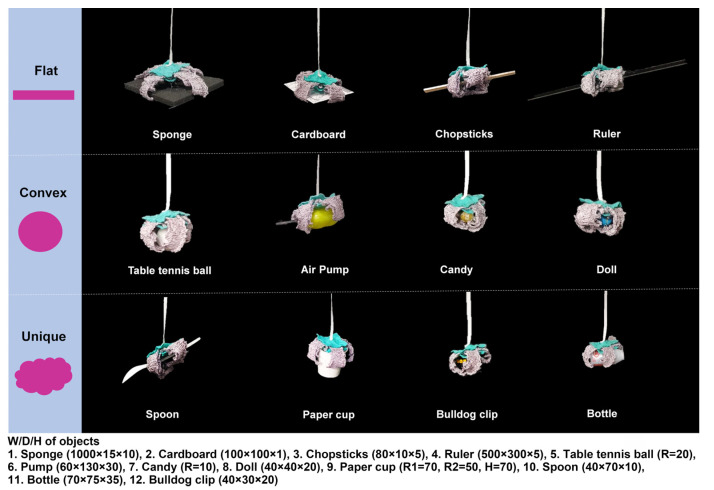
Gripping performance of the soft morphing textile gripper. The gripping force is maintained when a flat (Video S3), convex (Video S4), or unusually shaped object (Video S5) is held and moved up and down.

In Figure 10, the gripper finger was placed close to a large piece of cotton, and current was then applied. Under heating, the four fingers gripped the cotton, and the force was constant while the cotton was lifted (Video S2). This implies that the gripper has sufficient inward bending power to lift light, soft, and bulky objects. Furthermore, we checked if the gripper could hold and lift rigid and small objects such as a table tennis ball, which is an important factor for practical use. In this test, we prepared a series of daily objects with various shapes and sizes. Figure 11 shows that the gripper successfully grasped the given objects without wrinkling or damaging them. Various objects, including flat ones (Video S3), convex ones (Video S4), and unusually shaped ones (Video S5), were successfully moved up and down by the gripper.

## 5. Discussion and Conclusions

In this study, we proposed a soft morphing actuator based on loop-linked structures with functional fibers, which deforms under heating. The actuator was produced by combining active fiber and inactive fiber with the knitting technique. The active fiber was comprised of an SMA wire wrapped with polyester fiber, and the inactive fiber was conventional knitting yarn. The SMA is a unique intelligent material with a shape-memorizing ability as well as excellent mechanical properties. When the SMA is heated over its austenite start temperature, it deforms into its trained shape. The SMA wire in the knitted SMA actuator was trained to shorten under high temperatures, which makes the actuator bend while the SMA wire interacts with adjacent fibers.

After establishing the materials and production method, we investigated the structural/driving configurations to find the proper driving conditions. For this purpose, we varied the configurations and observed which ones allow for the maximum bending angle. Firstly, we determined the course-to-wale ratio. Five specimens (with ratios of 0.5, 0.8, 1.0, 1.2, and 1.8) were made and operated under the same current input. The end-tip angle was recorded, and the actuator with a ratio of 1.0 showed the largest bending angle. Secondly, we made three specimens with different numbers of bundled SMA wires (1–3) with the same course-to-wale ratio (1.0). Then, we monitored the exerting force of the actuators with a load cell. The actuator with three SMA wires exerted the largest force, which shows that when more SMA wires are used, the exerting force is larger. Thirdly, we determined the electric power input, while setting the course-to-wale ratio to 1.0 and using three strands of bundled SMA wires. The current flow was varied from 0 A to 0.16 A, and the actuator’s deformation was recorded during its actuation. At 0.06 A, the maximum end-tip deflection angle was only 4 degrees, which is insufficient to be used as an actuator. However, at 0.08 A, the value was 107 degrees, which is suitable for gripping objects. Over 0.08 A, there was no discernible difference in the deformation angle (within 1.5%). As a result, a minimum current of 0.08 A is needed to bend the actuator more than 100 degrees. Table 2 shows the maximum end-tip angle per unit length comparison between the proposed work and previous studies, which demonstrates that the knitted SMA wire has the greatest maximum end-tip angle among SMA-driven actuators, while possessing the second-greatest among all works.

After investigating the configurations of the textile actuator, we proposed an application as a soft morphing textile gripper. Three textile actuators were stitched together to make up each finger of the gripper. However, the single-layered textile actuator’s bending angle was insufficient for grasping an object. To solve this problem, we conducted a layer placement study. We made gripper fingers with different numbers of layers (1–3) and monitored the gripping angle and exerting force. As a result, the multi-layered structure showed a better curvature for object-grasping, while the three-layered textile actuator showed the largest exerting force, which demonstrates that a multi-layered structure is suitable for application as a gripper. To verify the soft morphing textile gripper’s applicability, we conducted a gripping performance test. In the test, the gripper was able to successfully lift flat/spherical/uniquely shaped objects, which shows its potential to be utilized as a gripper for handling fragile objects.

The proposed work is different from previous studies in that we focused not only on generating deformation but also comprehensively on the structural and driving configurations of the knitted textile actuator. It should be noted that this configuration study can be used as a reference and foundation for future research into the control of textile actuators. In addition, in the near future, it is envisaged that this fundamental study can be used in the development of wearable robots and exoskeletons by enhancing the strength, holding force, and accuracy of textile actuators.

## Figures and Tables

**Table 1:** Material properties of SMA wires.

Parameter	Unit	Value
$\mathrm{Martensitic}\;\mathrm{modulus}\;[E_{M}$]	GPa	26.3
$\mathrm{Austenitic}\;\mathrm{modulus}\;[E_{A}$]	GPa	75
Thermal coefficient [θ]	GPa	0.55
$\mathrm{Martensite}\;\mathrm{start}\;\mathrm{temperature}\;[M_{S}$]	°C	42
$\mathrm{Martensite}\;\mathrm{final}\;\mathrm{temperature}\;[M_{f}$]	°C	52
$\mathrm{Austenite}\;\mathrm{start}\;\mathrm{temperature}\;[A_{s}$]	°C	68
$\mathrm{Austenite}\;\mathrm{final}\;\mathrm{temperature}\;[A_{f}$]	°C	78
$\mathrm{Stress}\;\mathrm{influence}\;\mathrm{coefficient}\;[C_{M}$] (austenite to martensite)	MPa/°C	12
$\mathrm{Stress}\;\mathrm{influence}\;\mathrm{coefficient}\;[C_{A}$] (martensite to austenite)	MPa/°C	12
Initial martensite fraction of SMA	-	1.0
Diameter of wire	μm	200

**Table 2:** Maximum end-tip angle per unit length comparison between previous studies.

Types	Max. End-Tip Angle per Unit Length (°/mm)	Inventors/ Researchers
Straight-lined SMA wire	1.5	Lee et al. [43]
Straight-lined SMA wire	2.7	Lee et al. [44]
Straight-lined SMA wire	3.3	Song et al. [45]
Straight-lined SMA wire	2.1	Dezaki et al. [46]
Knitted SMA wire	3.9	This paper
Pneumatic chamber	0.8	Sun et al. [47]
Pneumatic chamber	0.4	Wang et al. [48]
Pneumatic chamber	1.3	Hao et al. [49]
Pneumatic chamber	0.5	Guo et al. [50]
Pneumatic chamber	0.7	Al-lbadi et al. [51]
Ferrofluid impregnated paper	4.3	Ding et al. [52]

## Supplementary Materials

The following supporting information can be downloaded at https://www.mdpi.com/article/10.3390/s23031518/s1. Video S1: Stepwise actuation of a gripper finger; Video S2: Demonstration of the gripping actuation (cotton); Video S3: Demonstration of the gripping actuation (flat shape); Video S4: Demonstration of the gripping actuation (convex shape); Video S5: Demonstration of the gripping actuation (unusual shape).
